# Prevention of posterior capsular opacification through cyclooxygenase-2 inhibition

**Published:** 2007-04-30

**Authors:** Heather L. Chandler, Curtis A Barden, Ping Lu, Donna F. Kusewitt, Carmen M. H. Colitz

**Affiliations:** The Ohio State University, Veterinary Biosciences, Columbus, OH

## Abstract

**Purpose:**

To determine if cyclooxygenase-2 (COX-2) is upregulated when lens epithelial cells (LEC) in clinical samples of cataracts and posterior capsule opacification (PCO) undergo epithelial-mesenchymal transition (EMT)-like changes. We also wanted to learn if inhibition of the enzymatic activity of COX-2 could prevent PCO formation.

**Methods:**

To ensure that EMT-like changes were occurring in LEC, real-time RT-PCR was used to examine expression of EMT markers. Clinical samples of canine cataracts and PCO were examined for COX-2 expression using immunohistochemistry, western blot analysis, and real-time RT-PCR. The COX-2 inhibitors, rofecoxib and celecoxib, were used in an ex vivo model of PCO formation, and the effects on cellular migration, proliferation, and apoptosis were analyzed using immunohistochemistry and western blots. Prostaglandin E2 (PGE2) expression was examined with ELISA.

**Results:**

Markers of EMT, such as lumican, Snail, Slug, and COX-2 were expressed in LEC. In clinical samples of cataracts and PCO, there was overexpression of COX-2 protein and mRNA. Both rofecoxib and celecoxib were effective at inhibiting PCO formation in our ex vivo model. Prevention of PCO with the COX-2 inhibitors appeared to work through decreased migration and proliferation, and increased apoptosis. Neither of the drugs had a toxic effect on confluent LEC and appeared to inhibit PCO through their pharmacologic action. Synthesis of PGE2 was inhibiting in the capsules treated with the COX-2 inhibiting drugs.

**Conclusions:**

Extracapsular phacoemulsification cataract surgery is the most common surgical procedure performed in human and veterinary ophthalmology. The most frequent postoperative complication is PCO. The LEC that remain adhered to the lens capsule undergo EMT-like changes, proliferate, and migrate across the posterior lens capsule causing opacities. We have shown that COX-2, a protein associated with EMT, is upregulated in canine cataracts and PCO. Inhibiting the enzymatic activity effectively prevented EMT of LEC in our ex vivo model of PCO through pharmacologic action, and not acute toxicity. These findings indicate that using COX-2 inhibitors in vivo may be an effective technique in preventing PCO.

## Introduction

Cataract, defined as an opacity of the lens or lens capsule, is the most common cause of visual impairment in dogs and humans [1,2]. Phacoemulsification extracapsular cataract extraction with intraocular lens (IOL) implantation is the most frequently performed ophthalmic surgical procedure in veterinary and human medicine, with a success rate of greater than 95% in both species [2,3]. The most common long-term complication following cataract surgery in both species is posterior capsule opacification (PCO) [4]. It is well established that, postoperatively, the primary response of the remaining anterior lens epithelial cells (LEC) is to undergo epithelial-mesenchymal transition (EMT)-like changes [5-7]. This results in posterior migration and proliferation of the LEC with subsequent vision impairment. EMT refers to a change in phenotype from an epithelial to fibrocytic morphology accompanied by aberrant basement membrane synthesis [4-7]. Hallmarks of EMT include the expression of cyclooxygenase-2 (COX-2), α-smooth muscle actin (α-SMA), lumican, and the transcriptional repressors Slug and Snail [5,8-10].

PCO-induced decreased visual acuity occurs in up to 50% of human adults following phacoemulsification surgery, with a higher risk in younger patients [11-16]. In addition, 100% of dogs that undergo phacoemulsification cataract surgery develop PCO within one year postoperatively [2,17]. The incidence of PCO in humans has been somewhat lowered, but not eradicated, by improvements in IOL design, such as the square edge. Similar IOL implantations are now being used in veterinary patients, and PCO is less severe in these dogs (personal observation, CMHC). In humans, PCO can be treated effectively with neodymimum:yttrium-aluminum-garnet (Nd:YAG) laser capsulotomy. However, the cost is substantial, and there can be significant morbidity due to postoperative complications including damage to the IOL, cystoid macular edema, retinal detachment, IOL subluxation, exacerbation of localized endophthalmitis, and retinal detachment [12]. In addition, YAG laser capsulotomy has not proven successful in canine patients due to their thicker posterior lens capsules. The development of alternative methods to prevent PCO is therefore of critical importance, and a pharmacologic method of inhibiting LEC EMT and proliferation would contribute markedly to the success of extracapsular cataract extraction with IOL placement.

There are three methods commonly used to deliver pharmacologic reagents to LECs after cataract surgery: (1) direct injection into the anterior chamber (with or without use of the *Perfect Capsule* device); (2) addition to irrigating solutions; or (3) impregnation of the IOL. The major difficulty with any drug delivery system is toxicity to other tissues, especially the corneal endothelium [18]. A number of pharmacologic agents have been evaluated for the prevention of PCO formation. Hypoosmolar agents and antimetabolites, such as catalin, methotrexate, mitomycin, and 5-fluorouracil, have been shown to lyse LEC and to be effective in inhibiting PCO in vitro [19,20]. Unfortunately, in vivo concentrations high enough to inhibit LEC proliferation, whether delivered as a single application or in a sustained release form, have resulted in toxicity to corneal endothelial cells, irides, ciliary body epithelial cells, and retina [19]. Topical antiinflammatory medications have also been evaluated for efficacy in the prevention of PCO. The nonsteroidal antiinflammatory drug (NSAID) diclofenac has been shown to inhibit proliferation of subconfluent cultures of LEC in a dose-dependent manner [21]. In vivo, topical diclofenac used three times daily for one year in human patients significantly reduced the incidence of PCO [22]. The use of NSAIDS and corticosteroids for only a month following cataract surgery, however, does not significantly inhibit PCO formation. Based on these findings, further evaluation of NSAIDS to inhibit PCO in dogs seems warranted.

COX-2 is an immediate early gene induced by a variety of stimuli, including cytokines, hormones, mitogens, and growth factors [23-27]. Increasing evidence links overexpression of COX-2 to EMT during embryonic development, fetal implantation, and tumor formation and invasion [8,28-30]. COX-2 and its metabolic products, such as prostaglandin E2 (PGE2), induce inflammation, activate antiapoptotic and anticell proliferation signaling pathways, and play a role in carcinogenesis and EMT [8,26,27,30,31]. Selective COX-2 inhibitors like celecoxib have been shown to be effective in chemoprevention of tumors of epithelial origin [32-34]. Although several mechanisms are proposed to explain the antitumor action of COX-2 inhibitors in epithelial cancers, the effects of these inhibitors on apoptosis and cell proliferation have been gaining attention [35,36]. Recent studies demonstrated that the proapoptotic effect of celecoxib is mediated by caspase-3 activation and that proliferation inhibition by celecoxib is due to downregulation of cyclins, resulting in cell cycle arrest [37,38].

In the present study, we evaluated expression of COX-2 in the normal canine lens and in LEC that have undergone EMT. We observed increased COX-2 expression in clinical samples of cataracts and in an ex vivo model of PCO. Specific COX-2 inhibitors effectively inhibited EMT of LEC in vitro by decreasing migration and proliferation and increasing apoptosis. We further demonstrated that the effects of COX-2 inhibitors were due to pharmacological activity rather than to acute toxicity. These findings suggest that COX-2 inhibitors may effectively eliminate postsurgical PCO in the dog.

## Methods

### Samples

Normal eyes were obtained by enucleation from dogs in good general health that were humanely euthanized at a local animal shelter for population control purposes. All dogs used in this study were estimated to be between 1 and 8 years of age, based on dentition and thickness of the anterior lens capsule. Globes were collected within 1 h of death and placed in 2% betadine solution, then rinsed and immersed in 1X phosphate buffered saline solution (PBS, pH 7.2) until dissection, which was performed within 2 h of enucleation.

### Normal whole lenses

Whole lens capsules were dissected using a method described in reference [39], then immediately fixed in neutral-buffered 10% formalin, paraffin embedded, and sectioned. Routine hematoxylin and eosin (H&E) staining was performed to assess the orientation of the specimens; 12 samples were selected for immunohistochemical staining and 24 samples were frozen, either for protein extraction (n=12) or RNA extraction (n=12).

### Primary canine lens epithelial cell cultures

Anterior lens capsules with adherent LEC were incubated in trypsin (1X EDTA, Gibco, Carlsbad, CA) for 5 min at 37 °C, and the supernatants were centrifuged to pellet LEC; LEC were then resuspended in DMEM (Gibco) and cultured in laminin-coated flasks (Beckton Dickinson, Franklin Lakes, NJ). LEC were examined daily to evaluate viability. Just prior to reaching confluence, they were replated.

### Anterior capsules with lens epithelial cells undergoing epithelial-mesenchymal transition

Anterior lens capsulotomy specimens were acquired from dogs with naturally occurring cataracts prior to routine phacoemulsification cataract extraction. Samples were obtained during continuous curvilinear capsulorrhexis and placed in 10% neutral-buffered formalin (n=20) or snap frozen and stored at -70 °C until protein (n=20) or RNA extraction (n=30) could be performed. Six whole cataractous lenses from canine patients with anterior lens luxations and three enucleated globes with naturally occurring PCO due to previous cataract surgery were fixed in 10% neutral-buffered formalin. Fixed lens samples were embedded in paraffin, sectioned at 4-5 μm, and stained with H&E. Samples were examined by light microscopy to determine if there were sufficient cells to allow immunohistochemical staining. For immunohistochemical staining, suitable sections were mounted onto charged slides (ProbeOn Plus, Fisher Scientific, Pittsburgh, PA).

### Sham cataract surgery and lens capsule harvesting

Freshly enucleated canine globes were used for this procedure. A 22 gauge needle was employed to begin the routine continuous curvilinear capsulorrhexis, which was performed using Utrada forceps. The lens nucleus and cortex were removed intact through the capsulotomy site and an irrigation/aspiration cannula was used to remove residual cortex from the lens capsule. The lenticular zonules were severed using Vannas scissors, and the lens capsule was gently excised from its vitreal attachments. The lens capsule was then placed in a sterile petri dish with the capsulotomy side up and pinned into place using four evenly spaced entomology pins. Five ml of DMEM containing antibiotic and antimycotic (Gibco, Carlsbad, CA) was added to the cavity formed by the immobilized lens capsule.

### Scratch model to induce epithelial-mesenchymal transition-like changes

LEC cultures were allowed to grow to 90% confluence in unsupplemented DMEM in a six-well laminin coated culture dish before a 1 mm scratch was made on the cellular surface. Cells were allowed to recover for 1, 2, 4, 8, or 24 h before being harvested for RNA isolation.

### Drug treatments

Celecoxib and rofecoxib were generously provided by Drs. C.-S. Chen and S. Kulp at The Ohio State University, College of Pharmacy. Both drugs were dissolved in DMSO then diluted to final working concentrations of 10 and 20 μM, respectively, in DMEM. To determine if the drugs could prevent EMT-like changes in vitro, we plated LEC cultures in a six-well laminin-coated culture dish. Cultures were grown in unsupplemented DMEM and treated with 10 or 20 μM rofecoxib, celecoxib, or vehicle and allowed to reach to 90% confluence. Six culture wells were treated with each drug dose. A 1 mm scratch was created and cells were allowed to recover for 24 h before being harvested for protein isolation. Digital images were taken immediately following the scratch and 24 h following wound healing. Images were uploaded and migration was quantified using Adobe Photoshop Version 6.0.

To determine if the drugs could prevent PCO, capsules were harvested as described in the previous paragraph and treated immediately. Every third day, the capsules (n=6) were given fresh media and drug. Control capsules received fresh media with vehicle (DMSO) only. LEC growth on capsules was monitored by digital photography every other day. Following 14 days, capsules were dissected into two pieces: Half of the pieces were placed in 10% neutral-buffered formalin and half were frozen for protein extraction. Histopathology with routine H&E staining was performed on half of the lens capsule. Cells still attached to the capsule were counted on the H&E slides.

To determine if LEC could recover the ability to undergo EMT following treatment with celecoxib or rofecoxib, we immediately treated six capsules with 20 μM celecoxib or rofecoxib following dissection. After seven days, treatment medium was replaced with medium containing vehicle only for the following seven days. Capsule morphology was monitored using phase contrast microscopy and photomicrographs. After 14 days, capsules were dissected into two pieces: Half went into 10% neutral-buffered formalin for subsequent histology, and half were frozen for protein extraction.

To determine if the drugs were acutely toxic to LEC, capsules were treated with vehicle for the first seven days. Afterwards, the capsules were treated with 20 μM celecoxib or rofecoxib for seven days. Capsules were monitored by phase contrast microscopy and digital photomicrography every other day. After 14 days, capsules were dissected into two pieces; half of these were placed in 10% neutral-buffered formalin, and half were frozen for protein extraction. LEC were examined for markers of apoptosis using immunohistochemistry against cleaved caspase-3 and TUNEL staining.

### Antibodies

Immunohistochemical staining was performed using antibodies against COX-2 (1:200; Santa Cruz Biotechnology, Santa Cruz, CA), cleaved caspase-3 (1:20; Oncogene, San Diego, CA), α-SMA (1:100; Spring Bioscience, Freemont, CA), and PCNA (1:200; DAKO Corp., Carpinteria, CA). Secondary antibodies included, as appropriate, antigoat (Vector Laboratories, Burlingame, CA), antirabbit (Biogenex Super Sensitive Link; Biogenex, San Ramon, CA), and antimouse (Vector) antibodies. DAKO Antibody Diluent with Background Reducing Components (DAKO) was used to dilute antibodies.

### Immunohistochemical staining

Standard avidin-biotin complex (ABC) immunohistochemistry was performed. Briefly, decerated paraffin-embedded sections were incubated with antigen retrieval solution (DAKO) at 90 °C for 20 min and allowed to cool to room temperature for 20 min. All subsequent steps were performed at room temperature. Samples were treated with peroxidase block (DAKO) for 10 min, washed with 1X Tris buffered saline containing 0.1% Tween 20 (TBST), then incubated in protein block for 20 min (DAKO). Primary antibodies were applied to slides for 1 h at room temperature. After slides were washed in TBST, secondary antibodies were applied for 1 h at room temperature. Slides were washed in 1X TBST, then incubated in ABC (Vector Laboratories) for 1 h. Following washes in 1X TBST, immunostaining was then visualized with either diaminobenzidine tetrahydrochloride (DAB; DAKO) applied for 3 min at room temperature in the dark or with Vector NovaRED (Vector Laboratories) applied for 5 min at room temperature. Following washes, slides were counterstained with hematoxylin, dehydrated, and permanently mounted. Stained samples were visualized by light microscopy and photographed (Olympus; Melville, NY). Positive controls consisted of canine squamous cell carcinoma and canine mammary gland tumors. Negative controls omitted the primary antibody. Controls were performed concurrently with each batch of slides.

### TUNEL staining

Apoptosis was detected in treated capsules by the TUNEL assay (Apoptosis Detection System with fluorescein; Promega Corp., Madison, WI). Decerated paraffin-embedded sections were treated with proteinase K (20 mg/ml) for 10 min at room temperature, washed, fixed in 4% methanol-free formaldehyde solution for 5 min, and covered with equilibration buffer. Terminal deoxynucleotidyl transferase-buffer containing nucleotide mix was applied to the sections for 60 min at 37 °C in a moist chamber. The sections were washed and counterstained with propidium iodide.

### Immunoblot analysis

Whole cell protein was extracted from frozen normal canine anterior lens capsules and anterior capsulotomy samples from clinical canine cataracts using 1X CHAPs lysis buffer (Chemicon, Temecula, CA). Samples in CHAPs buffer were incubated on ice for 30 min, then centrifuged at 12,000x g for 20 min. Five μl of the resulting supernatant was used for quantification of protein concentration by Bradford protein assay (Bio-Rad, Hercules, CA); the remainder was stored at -80 °C until used.

Samples were denatured (95 °C, 3 min) in modified sodium dodecyl sulfate (SDS) sample loading buffer, and protein (15 μg) was separated by SDS/PAGE (10% acrylamide, v/v) at 150 V for approximately 1.5 h. After electrophoresis, proteins were transferred to a nitrocellulose membrane at 300 mA for 1.5 h. Nonspecific binding was blocked by incubating the membrane for 5 h at room temperature in 5% nonfat dry milk diluted in 1X TBST. After blocking, membranes were incubated overnight with a goat anti-COX-2 antibody (Santa Cruz) that had been diluted 1:500 in blocking solution. The membrane was then washed extensively (5x10 min) with 1X TBST, and secondary antibody (antigoat immunoglobin; Vector Laboratories) was added in blocking solution (1:10,000) for 1 h at room temperature. Protein was detected using the Pierce (Rockford, IL) Femto Immunoblotting system and Kodak X-OMAT AR film. Membranes were stripped (Restore stripping solution, Pierce) and the technique was repeated using antibeta-actin antibody (1:5000; Sigma, St. Louis, MO) as a loading control.

### Quantitative RT-PCR

Total RNA was extracted using Absolutely RNA Microprep Kit (Stratagene, La Jolla, CA) as suggested by the manufacturer. The ImProm II Reverse Transcriptase kit (Promega) was used to synthesize the first strand cDNA as per the manufacturer's instructions. Quantitative real-time RT-PCR was performed in the Stratagene Mx3000p Multiplex Quantitation System as follows: 95 °C for 15 min, then 40 cycles of 94 °C for 30 s, 60 °C for 30 s, and 72 °C for 30 s, using the QuantiTect SYBR Green PCR kit (Stratagene). Primers to amplify sequences of the canine *COX-2* and *HPRT* (housekeeping control) genes were designed based on cloning and sequence data obtained in our laboratory. The primers used are presented in Table 1.

**Table 1:** Primers used to amplify sequences of the canine COX-2 and HPRT (housekeeping control) genes.

Name	Sequence
COX-2	F: GCTTCGATTGACCAGAGCAG
COX-2	R: CACCATAAAGGGCCTCCAAC
Lumican	F: CCTGGAGGTCAATGAACTCG
Lumican	R: TGTGGGTAAGACGGTTGCCA
Slug	F: CCCTGAAGATGCATATTCGGAC
Slug	R: CTTCTCCCCCGTGTGAGTTCTA
Snail	F: CGGAAGCCTAACTACAGCGA
Snail	R: GGACAGAGTCCCAGATGAGC
HPRT	F: TGACACTGGTAAAACAATG
HPRT	R: GGTCCTTTTCACCAGCAAGCT

The sequences were designed based on cloning and sequence data obtained in our laboratory.

Real time RT-PCR was repeated 3 times on each sample. The relative amounts of COX-2 mRNA, normalized to HPRT levels, were calculated according to the method described by Ramakers [40]. Results were expressed as the ratio of target gene to HPRT gene expression.

### Quantitation of PGE2 levels

Tissue culture media was collected from lens capsules treated with vehicle, rofecoxib, or celecoxib and snap-frozen in liquid nitrogen. PGE2 levels were assessed using the PGE2 ELISA kit from Cayman Chemical (Ann Arbor, MI) according to the manufacturer's instructions.

## Results

### Markers of epithelial-mesenchymal transition are expressed in lens epithelial cells

**Figure 1:**
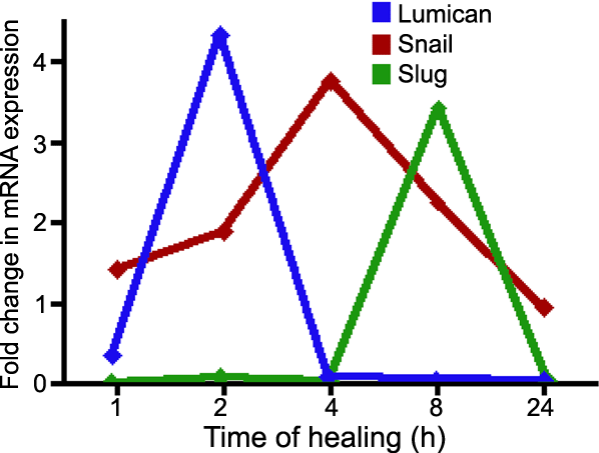
Expression of markers of EMT in LEC. A 1 mm scratch was created in confluent LEC and allowed to heal before real-time RT-PCR was used to determine expression. Lumican is upregulated early in EMT and expression peaks by 2 h before returning to basal expression by 4 h. Expression of the Snail in LEC peaks by 4 h and Slug expression peaks by 8 h. Both Snail and Slug return to basal expression by 24 h.

Using real-time RT-PCR, we examined the expression of markers of EMT-like changes in our canine LEC scratch model. Lumican expression peaked 2 h following the wounding event and returned to basal expression by 4 h (Figure 1). By 4 h, Snail expression peaked and reached basal expression in 24 h (Figure 1). Slug expression remained at basal levels until peaking at 8 h before decreasing expression at 24 h (Figure 1). In support of other studies, this data provides evidence that EMT-like changes occur in canine LEC.

### Cyclooxygenase-2 expression in the canine lens

**Figure 2:**
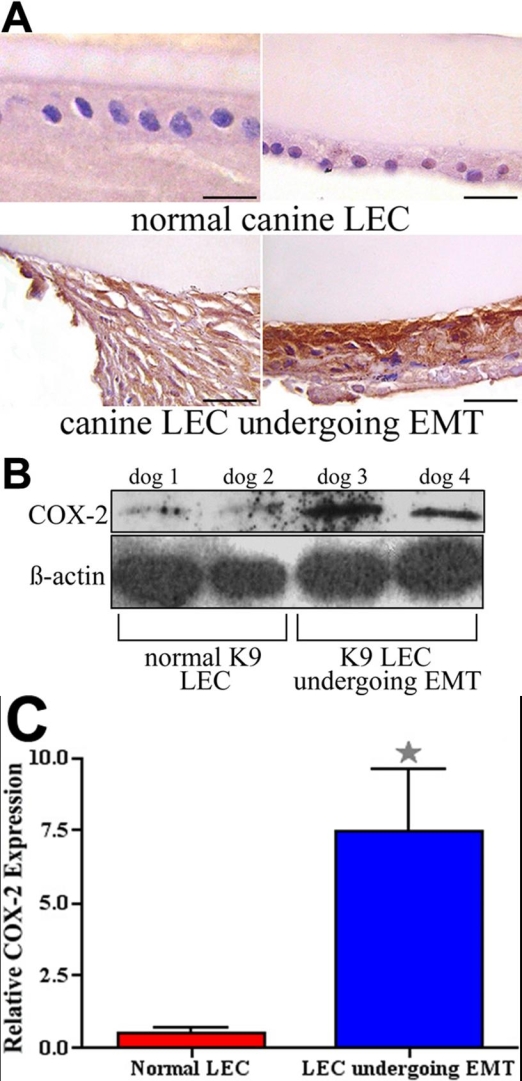
Expression of COX-2 in normal canine LEC and in LEC undergoing EMT-like changes. **A**: In normal LEC there is little to no COX-2 immunoreactivity, while LECs in cataractous and PCO sample show marked cytoplasmic staining for COX-2, as indicated by diffuse brown cytoplasmic staining. The scale bar is equal to 30 μm. **B**: Western blot analysis of COX-2 in normal canine LEC and in LEC undergoing EMT-like changes. In normal LEC (dog 1 and 2) there is little COX-2 protein present, while in clinical samples of cataract and PCO (dog 3 and 4) there is increased expression of COX-2 protein. **C**: Using real-time RT-PCR, expression of COX-2 mRNA was analyzed. In normal LEC there is some expression of COX-2 mRNA, while in clinical samples of cataract and PCO there is a significant (p<0.0145) upregulation of COX-2. Error bars indicate SEM.

There was minimal COX-2 immunoreactivity in the normal dog eye, and this was restricted to the epithelium of the ciliary body. The LEC of canine cataractous capsulotomy and PCO samples had diffuse cytoplasmic COX-2 immunoreactivity, while the LEC of normal canine lenses were COX-2-negative (Figure 2A). Western immunoblotting and quantitative RT-PCR confirmed that LEC from clinical cataract samples had increased COX-2 protein and mRNA expression compared to normal LEC (Figure 2B,C). Normal LEC expressed significantly lower (p<0.0145) levels of COX-2 mRNA and protein than cataractous LEC. Taken together, these results indicated COX-2 expression was upregulated in LEC undergoing EMT.

### Effects of cyclooxygenase-2 inhibitors on epithelial-mesenchymal transition of lens epithelial cells

**Figure 3:**
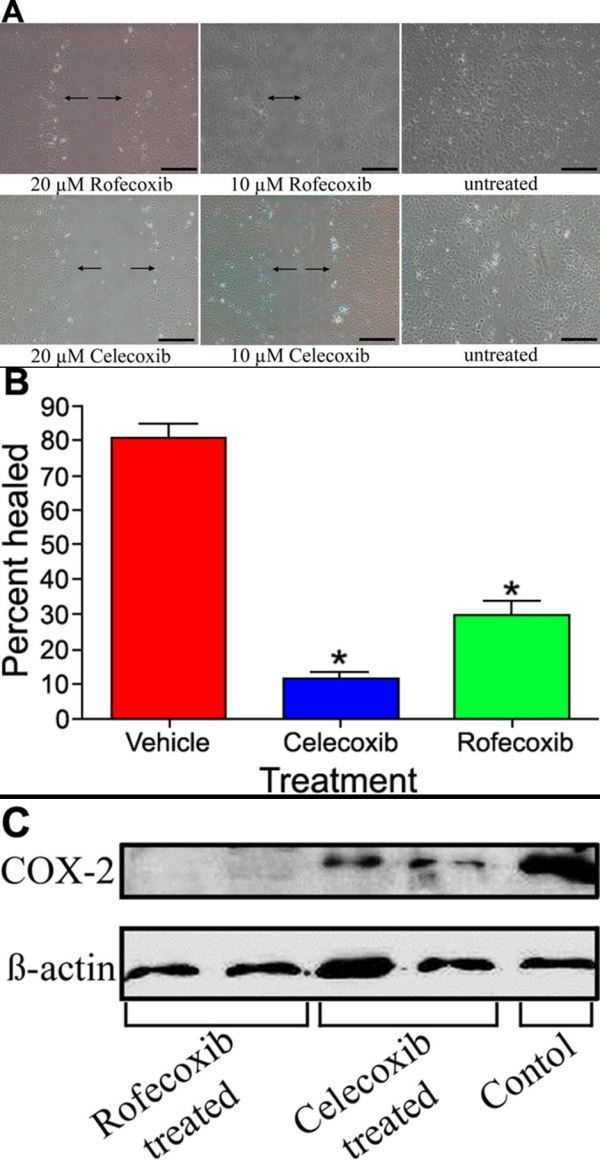
Effects of rofecoxib and celecoxib in migrating LEC in vitro. **A**: Cells were treated with 10 or 20 μM rofecoxib, celecoxib, or vehicle before a 1 mm scratch was created and LEC were allowed to heal for 24 h. Both rofecoxib and celecoxib treated LEC had decreased rates of wound closure compared to the control. There was a dose dependent response to drug treatment. The scale bar is equal to 0.5 mm. **B**: There was significantly (p<0.001) decreased healing in LEC cultures treated with either celecoxib or rofecoxib compared to controls. **C**: Western blot analysis of the LEC treated with 20 μM rofecoxib or celecoxib. There is increased expression of COX-2 in control LEC undergoing EMT-like changes, and decreased expression in LEC treated with the COX-2 inhibitors. There is no expression of COX-2 in rofecoxib treated cells while there is still some COX-2 protein present in the celecoxib treated cells.

To determine if COX-2 inhibition would prevent EMT-like changes in LEC, we allowed primary cultures of normal canine LEC to grow to 95% confluence. A 1 mm scratch wound was then created and cultures were treated with either vehicle, 10 μM rofecoxib or celecoxib, or 20 μM rofecoxib or celecoxib. After 24 h, wounds in the vehicle-treated cultures had closed, while the wounds in the rofecoxib and the celecoxib-treated cultures remained open (Figure 3A). There was a significant (p<0.001) delay in LEC migration in both the celecoxib and rofecoxib treatments compared to the controls (Figure 3B). Immunoblotting revealed that COX-2 expression was decreased in the rofecoxib- and celecoxib-treated cultures compared to vehicle-treated cultures (Figure 3C). Rofecoxib appeared to inhibit COX-2 expression more effectively than celecoxib.

### Effects of cyclooxygenase-2 inhibitors on posterior capsule opacification formation

**Figure 4:**
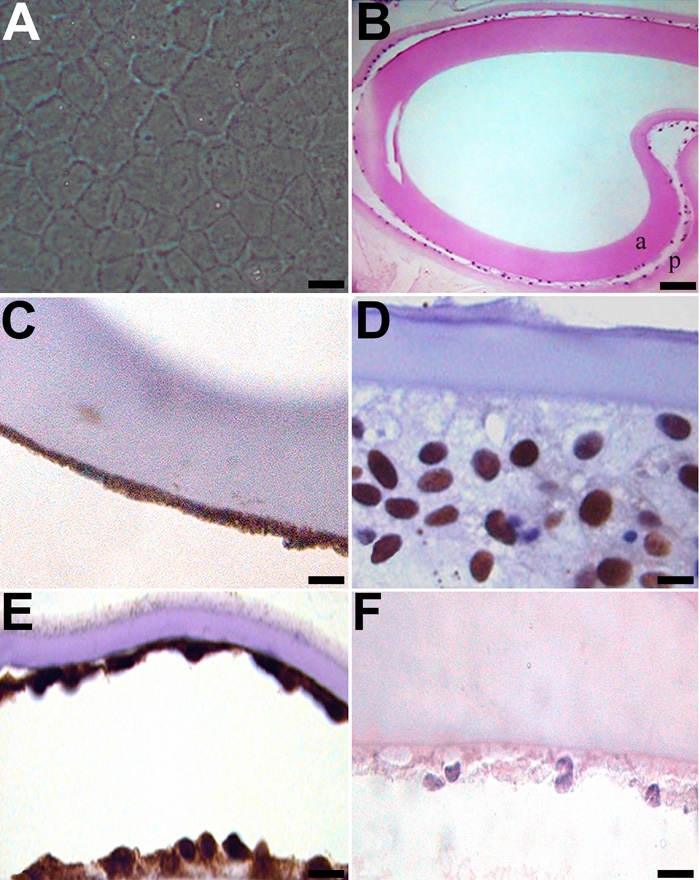
EMT-like changes in the ex vivo lens capsule model. Following sham cataract surgery, canine lens capsules with residual LEC present on the anterior lens capsule were treated with vehicle alone for 2 weeks. By the end of this period, LEC were present on the anterior capsule (a) and had migrated across the posterior lens capsules (p; **A** and **B**). These LEC were immunopositive for COX-2, PCNA, and α-SMA (**C**-**E**, respectively). To evaluate apoptosis, capsules were stained for cleaved caspase-3 (**F**). The scale bars are equal to 20 μm, except for **B**, where the scale bar is equal to 50 μm.

Following sham cataract surgery, canine lens capsules with residual LEC present on the anterior lens capsule were treated with vehicle alone for 2 weeks. By the end of this period, LEC had migrated across the posterior lens capsules (Figure 4A,B). These LEC were immunopositive for COX-2, PCNA, and α-SMA, indicating that the cells were both migrating and proliferating (Figure 4C-E). There was minimal immunoreactivity for cleaved caspase-3, indicating that LEC were not undergoing apoptosis (Figure 4F). The absence of TUNEL positivity confirmed caspase-3 findings (data not shown). In addition, western immunoblot analysis revealed increased COX-2 expression. Changes seen in cultured canine LEC following sham cataract surgery were similar to the EMT changes seen in clinical cases of PCO formation. Thus our ex vivo model mimics clinical PCO formation.

**Figure 5:**
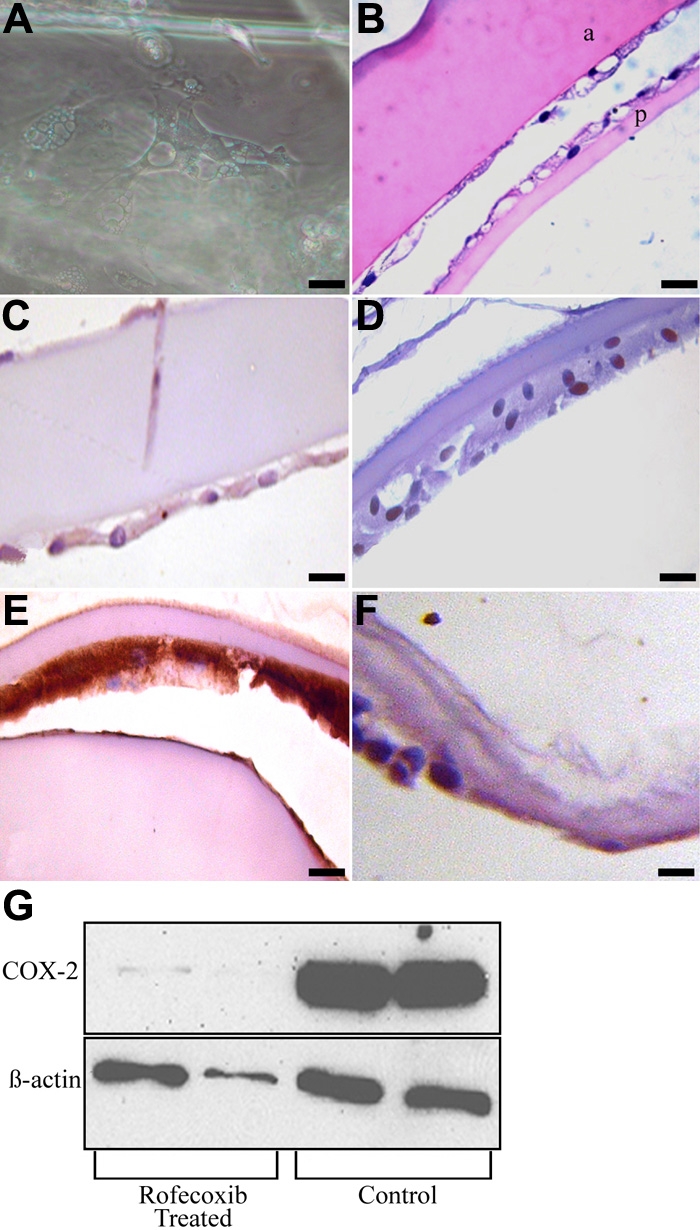
Effect of rofecoxib on EMT in LEC. In canine lens capsules treated for 2 weeks with 10 μM rofecoxib immediately following sham cataract surgery, remaining LEC had altered morphology and adhesion (**A** and **B**). Magnification 40X. "a" indicates the anterior capsule and "p" indicates the posterior capsule. Immunohistochemistry revealed little or no staining for COX-2, decreased expression of PCNA and α-SMA, and increased expression of cleaved caspase-3 (**C**-**F**, respectively). Bars, 20 μm. Western blot analysis of the LEC treated with 10 μM Rofecoxib following sham cataract surgery (**G**). COX-2 protein was decreased in rofecoxib-treated capsules compared to vehicle-treated capsules.

**Figure 6:**
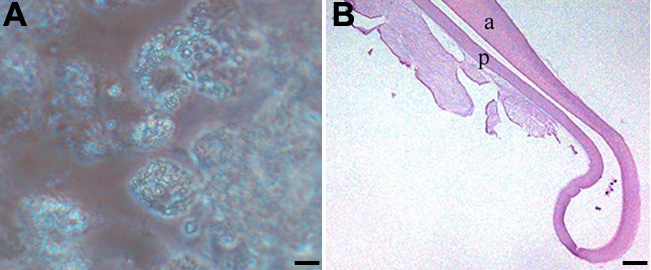
Effects of high doses of rofecoxib on EMT in LEC. There were very few LEC in lens capsules treated with 20 μM rofecoxib for 2 weeks. The LEC present were highly vacuolated and did not adhere to the adjacent basement membrane as seen in phase contrast microscopy (**A**, Bar, 20 μm) and histology (**B**, Bar 50 μm).

**Figure 7:**
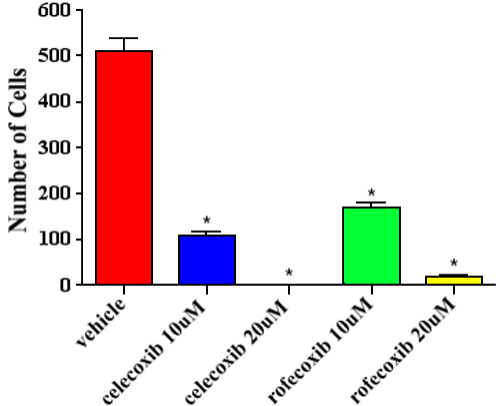
Number of cells adhered to the lens capsule following treatment with rofecoxib or celecoxib. Cells were counted from a hematoxylin and eosin stained slide of 6 different lens capsules in each treatment group. There was a significant decrease (p<0.001) in the number of cells present in the celecoxib and rofecoxib treated groups compared to the vehicle controls.

In canine lens capsules treated with 10 μM rofecoxib for two weeks immediately following sham cataract surgery, LEC were vacuolated and did not adhere normally to the underlying lens capsule (Figure 5A,B). Immunohistochemistry revealed little or no staining for COX-2, increased expression of cleaved caspase-3, and decreased expression of PCNA and α-SMA (Figure 5C-E). Results from the TUNEL assay confirmed the caspase-3 staining, revealing increased numbers of positive cells, both indicating increased apoptosis (data not shown). As determined by western immunoblot analysis, COX-2 protein was decreased in rofecoxib-treated capsules compared to vehicle-treated capsules (Figure 5G). There were few LEC in lens capsules treated with 20 μM rofecoxib for 2 weeks (Figure 6A,B). The LEC present were highly vacuolated and did not adhere to the adjacent basement membrane. Due to the low numbers of LEC in these samples, immunohistochemistry and western immunoblot analysis were not performed. The number of cells adherent to the lens capsule were counted from an H&E hematoxylin and eosin slide. There were significantly fewer cells (p<0.001) present in the rofecoxib-treated groups compared to the vehicle controls (Figure 7).

**Figure 8:**
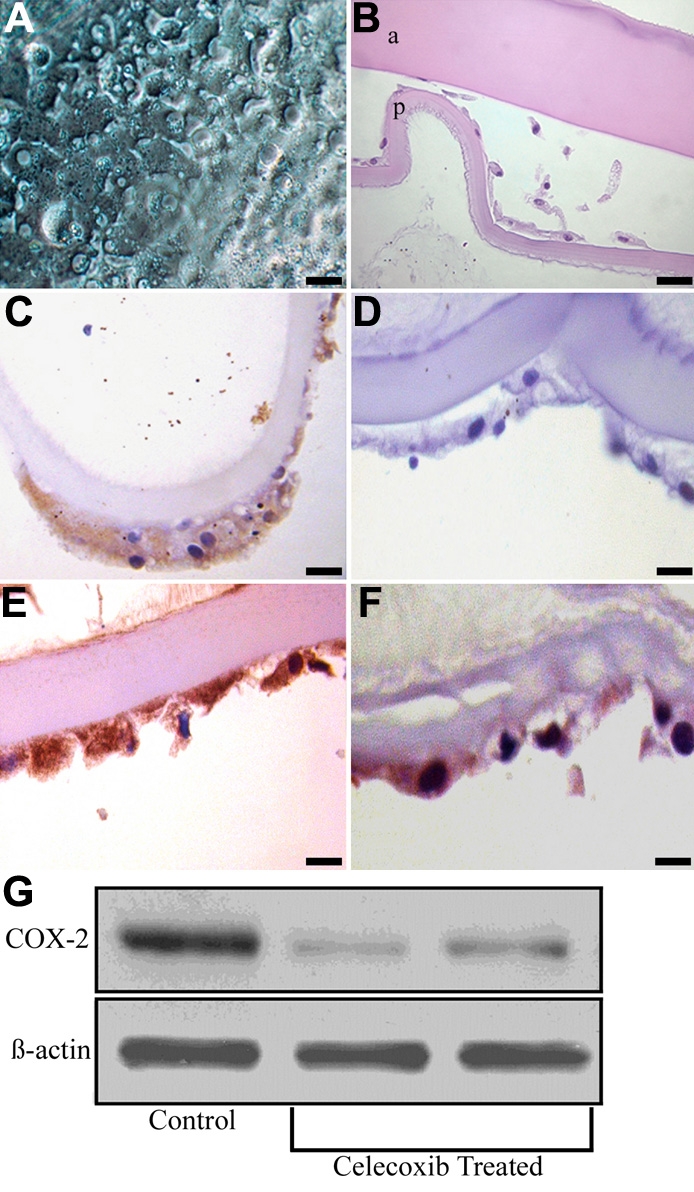
Effects of celecoxib on EMT in LEC. Following sham cataract surgery, canine lens capsules treated with 10 μM celecoxib for 2 weeks had some remaining LEC with altered morphology and adhesion (**A** and **B**). Magnification 40X. "a" indicates the anterior capsule and "p" indicates the posterior capsule. Immunohistochemical staining revealed that the remaining LEC expressed a moderate amount of COX-2, did not express PCNA, showed decreased α-SMA immunostaining, and were strongly immunoreactive for cleaved caspase-3 compared to untreated capsules (**C**-**F**, respectively). Bars, 20 μm. Western blot analysis of the LEC treated with 10 μM celecoxib following sham cataract surgery (**G**). COX-2 protein was decreased in celecoxib-treated capsules compared to vehicle-treated capsules.

**Figure 9:**
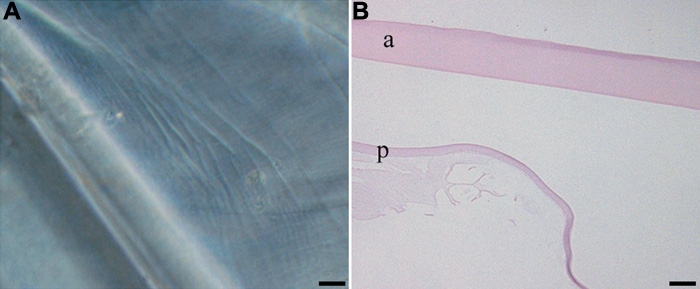
Effects of high doses of celecoxib on EMT in LEC. Lens capsules treated with 20 μM celecoxib for 2 weeks were devoid of all LEC on the anterior (a) and posterior (p) capsules as seen through phase contrast microscopy (**A**, the scale bar is equal to 20 μm) and histology (**B**, the scale bar is equal to 50 μm).

Following sham cataract surgery, canine lens capsules treated with 10 μM celecoxib for two weeks also had vacuolated LEC that did not adhere to the underlying basement membrane (Figure 8A,B). Immunohistochemical staining revealed that the remaining LEC (1) expressed a moderate amount of COX-2, (2) were strongly immunoreactive for cleaved caspase-3, (3) did not express PCNA, and (4) showed decreased α-SMA immunostaining compared to untreated capsules (Figure 8C-F). As with the rofecoxib-treated capsules, there was an increase in the number of TUNEL positive cells (data not shown). Western blotting confirmed COX-2 protein was decreased in the celecoxib-treated capsules compared to the vehicle-treated capsules (Figure 8G). Lens capsules treated with 20 μM celecoxib for two weeks were devoid of all LEC (Figure 9A,B). A count of cells still adhered to the lens capsule revealed a significant decrease (p<0.001) in the celecoxib-treated groups compared to the controls (Figure 7).

### Prostaglandin E2 expression following treatment with rofecoxib or celecoxib

**Figure 10:**
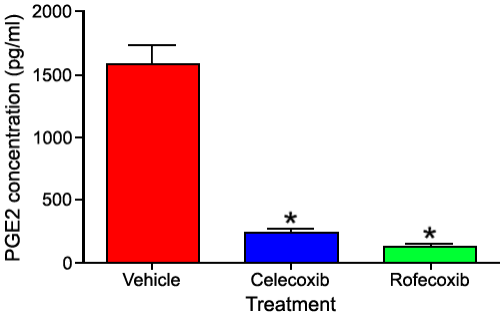
Expression of PGE2 in LEC treated with 10 μM rofecoxib or celecoxib for 2 weeks. Synthesis of PGE2 was significantly decreased (p<0.0001) in canine capsules that were treated with rofecoxib or celecoxib compared with vehicle-treated capsules.

To determine if COX-2 inhibition was reflected in reduced synthesis of its products, expression of PGE2 was evaluated. We found that synthesis of PGE2 was significantly decreased (p<0.0001) in canine capsules that were treated with rofecoxib or celecoxib compared with vehicle-treated capsules (Figure 10).

### Evaluating recoverability in treated lens epithelial cells

**Figure 11:**
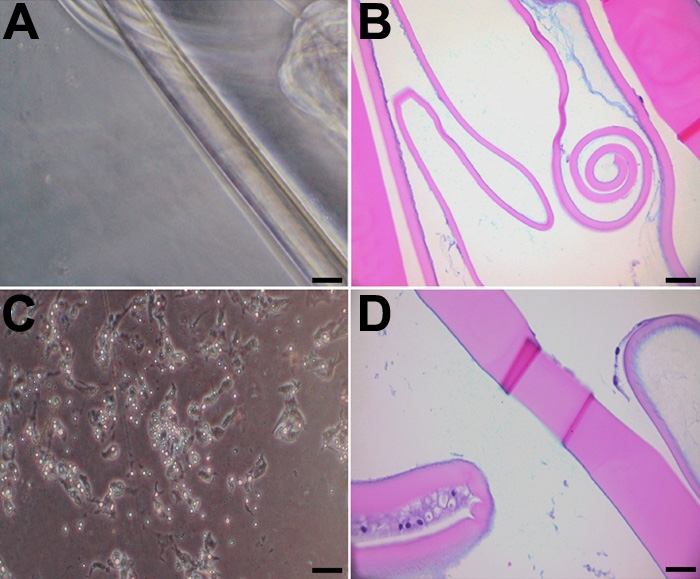
Evaluating the recoverability of LEC following COX-2 inhibition. LEC on capsules treated with drug for one week and were allowed to recover in medium containing vehicle only for a second week. LEC that were incubated in either celecoxib (**A** and **B**) or rofecoxib (**C** and **D**) during the first week of culture did not undergo EMT. The scale bars are equal to 30 μm (**A** and **C**) and to 50 μm (**B** and **D**).

**Figure 12:**
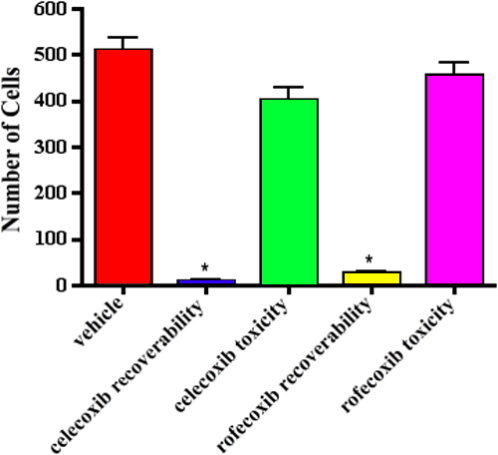
Cell counts to evaluate recoverability and toxicity following COX-2 inhibition. Cells were counted from a hematoxylin and eosin stained slide of 6 different lens capsules in each treatment group. There is a significant decrease (p<0.001) in the number of cells adhered to the lens capsule following treatment with the COX-2 inhibitors for week 1 before capsules were treated with vehicle for week 2. There was no significant decrease in the number of cells present in the capsules that received vehicle for week 1 and the COX-2 inhibitor for week 2 compared to the vehicle controls.

LEC on capsules treated with drug for one week were allowed to recover in vehicle-only medium for a second week. As shown previously, EMT was inhibited when LEC were incubated in either rofecoxib or celecoxib during the first week of culture. Because the capsules were treated with the drugs for only one week, as compared to two weeks in our previous experiments, some LEC remained on the capsule. This allowed us to determine if the LEC could repopulate the capsule. Moreover, even after one week of culture in the absence of drug, the LEC did not regain the ability to adhere to the lens capsule and proliferate and undergo EMT (Figure 11A-D). The number of cells adhered to the lens capsule were counted from a H&E slide. There was a significant decrease (p<0.001) in the number of cells present in either the rofecoxib- or celecoxib-treated groups compared to the vehicle controls (Figure 12).

### Evaluating the toxic effects of rofecoxib and celecoxib

**Figure 13:**
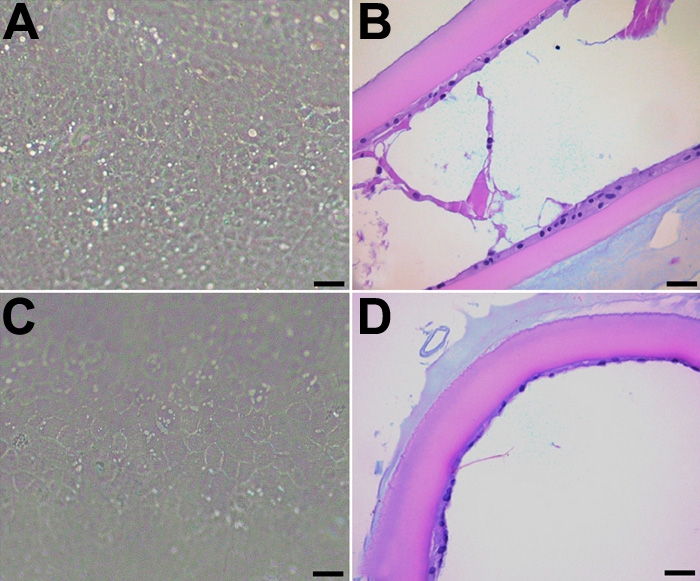
Evaluating the toxic effects of celecoxib and rofecoxib on LEC not undergoing EMT. To determine if the effects of rofecoxib or celecoxib were due to specific pharmacologic activity or to non-specific chemical toxicity, sham cataract surgery was performed and LEC were treated with vehicle only for one week, then treated with 20 μM celecoxib (**A** and **B**) or rofecoxib (**C** and **D**) for a second week. There were no signs of toxicity in the LEC. The scale bars are equal to 30 μm (**A** and **C**) and to 50 μm (**B** and **D**).

In an attempt to determine if the effects of rofecoxib or celecoxib were due to specific pharmacologic activity or to nonspecific chemical toxicity, we performed sham cataract surgery and treated LEC with vehicle only for one week, during which time EMT occurred as seen previously. Lens capsules were then treated with 20 μM rofecoxib or celecoxib for a second week. LEC in these samples did not exhibit any morphologic signs of nonspecific toxicity. After a week of culture in the presence of inhibitor, LEC remained adherent to the underlying capsule (Figure 13A-D) and were negative for cleaved caspase-3 and TUNEL (data not shown). A count of cells still adhering to the lens capsule revealed no significant decrease in the rofecoxib- or celecoxib-treated groups compared to the controls (Figure 12). These findings suggested that both drugs inhibited PCO through their pharmacologic actions rather than by non-specific toxicity. However, inhibition of EMT by the drugs appeared to be irreversible.

## Discussion

Remaining LEC on the anterior lens capsule following extracapsular lens extraction in rabbits and humans initially proliferate and, by four days after cataract surgery, undergo EMT [11,41]. LEC at the equatorial lens bow migrate posteriorly to populate the posterior capsule, a markedly abnormal location [12,42]. In ex vivo cultures of canine lens capsules, LEC migrate across the majority of the posterior lens capsule by 14 days following sham cataract surgery, a situation that mimics PCO formation in vivo [43]. The EMT that occurs during PCO formation appears to be initiated by signals originating from outside the epithelial cell, including extracellular matrix components such as collagen and soluble growth factors [44-46]. Activation of TGF-β and EGF signaling pathways are sufficient to induce EMT-like changes in LEC, and these pathways have been shown to be involved in cataractogenesis and PCO formation [18,47-51]. Injured LECs at capsulotomy edges show enhanced expression of EGF receptors, increasing their responsiveness to EGF in the aqueous humor [52]. Thus, growth factor-driven changes in cell phenotype may play an important role in the cell migration and EMT that characterize PCO.

Many factors in the cataract extraction process influence PCO severity, including capsulorrhexis technique, use of hydrodissection, composition of the irrigating solution, and type of intraocular lens prosthesis [12,17,53,54]. None of the currently used techniques of cataract surgery completely eliminates the risk of PCO formation. Nd:YAG laser posterior capsulotomy is the most commonly used therapeutic modality for treatment of PCO. Even minimal haze in humans may require secondary capsulotomy. This procedure cost the United States Medicare Program 250 million dollars in 1993 [55]. A 2001 study found that 3.6 billion dollars were spent on cataract-related eye care [56]. As well, younger individuals are predisposed to PCO development. A 1989 study determined that 70% of patients under 40 years old require a second Nd:YAG laser capsulotomy compared to 37% of patients older than 60 years [57]. In chronic PCO lesions, the mechanical forces of pseudometaplastic LEC contraction, caused by expression of α-SMA, can lead to striae or folds in the posterior capsule and to IOL decentration as well as anterior capsule contracture syndrome [12]. The capsulotomy procedure performed to treat PCO is not without its own potential complications, including damage to the IOL, intraocular pressure elevation, cystoid macular edema, retinal detachment, IOL luxation, and endophthalmitis [12].

COX-2 is an inducible enzyme normally undetectable in most cell types [58]. COX-2 is involved in the immediate-early gene response to cytokines and growth factors, such as EGF [59]. We have discovered cataractous LEC that have undergone EMT express COX-2 mRNA and protein, but normal LEC do not. An abundance of evidence now exists demonstrating a role for COX-2 in EMT. For example, one of the products of COX-2 activity, PGE2, has been shown to be a critical player mediating the development of cancer. PGE2 stimulates increased proliferation, altered adherence, increased migration, and enhanced invasiveness of cancer cells [60-63]. Our study demonstrated that inhibition of COX-2 with celecoxib and rofecoxib in an ex vivo model of PCO results in decreased PGE2 production by LEC and decreased proliferation and increased apoptosis of LEC.

Other studies have found that COX-2 prevention of proliferation was dose-dependent and mediated through inhibition of cell-cycle progression, particularly through tight regulation of the G_1_-S checkpoint, controlled by cyclin D_1_ and cyclin E [37]. While our study did not examine the specific mechanism through which celecoxib exerts its effects on cell-cycle regulation, we have provided evidence that EMT-like changes in LEC are inhibited by celecoxib at least in part through decreased cellular proliferation. In the present study, we also demonstrate that celecoxib induction of apoptosis results in increased expression of cleaved caspase-3. Several other studies support this finding and indicate that celecoxib-induced apoptosis may be mediated through activation of a caspase pathway [37,38]. Alternatively, celecoxib may promote apoptosis through the PDK1 pathway, with subsequent inhibition of Akt [36]. It has been reported that rofecoxib has no appreciable effects on Akt signaling in relation to apoptosis [64]. Our data suggest that celecoxib is a more potent inhibitor of PCO formation ex vivo, potentially through the increased Akt regulated apoptosis. Studies are currently being performed in our laboratory to evaluate the role of Akt in PCO formation. Because increased cell proliferation and resistance to apoptosis contribute to PCO formation, simultaneously counteracting multiple processes provides a more effective approach toward preventing secondary cataractogenesis.

The data presented here suggest that there is a feedback mechanism for the regulation of COX-2 expression. This is supported by other studies that have found a positive feedback cycle between two systems: one that involves COX-2 synthesis of PGE2, the other involving aromatase control over estrogen synthesis [65,66]. Although the mechanisms are not fully understood, estrogen can induce expression of COX-2 and subsequently of PGE2 through induction of various cytokines and growth factors. High levels of PGE2 can then induce aromatase activity, which controls estrogen synthesis [65,66]. Preliminary data obtained in our laboratory indicate that LEC undergoing EMT, have increased expression of estrogen and higher levels of aromatase activity compared to normal LEC [67]. This is one potential mechanism by which COX-2 expression may be controlled in canine cataract and PCO samples and further supports the use of COX-2 inhibitors to prevent EMT of LEC.

Intraocular application of pharmacologic agents to prevent PCO has been investigated by several authors with the goal of selectively destroying LEC while avoiding toxic side effects on other intraocular tissues. Corneal endothelial toxicity is a major concerns when intraocular drugs are used. Preliminary data from our laboratory indicate that neither celecoxib nor rofecoxib is toxic to corneal endothelial cells, based on cell morphology and the absence of caspase-3 staining. Moreover, because there is little basal expression of COX-2 within the normal eye, the use of COX-2 inhibitors to prevent PCO formation is unlikely to affect the eye adversely. Other studies support our present finding that COX-2 expression in the normal canine eye is restricted to low levels in the ciliary body epithelium [68-70]. In addition, the data presented here suggest that celecoxib and rofecoxib have few toxic effects on LEC not undergoing EMT, thus these drugs may be safe for nonmigrating cells. While we recognize that rapidly dividing cells may be more susceptible to toxicity than quiescent confluent cells, we believe that early treatment of LEC undergoing EMT provides a potentially effective yet safe treatment for PCO.

It remains to be determined if simply flushing the capsule during or immediately following cataract surgery with either celecoxib or rofecoxib will be sufficient to inhibit PCO formation or if extended drug release will be necessary. We are currently examining many options for incorporating COX-2 inhibitors into current PCO prevention strategies, including long-term topical application of celecoxib or rofecoxib and impregnating IOL with the drugs for release over time. Preliminary data are promising, and suggest that both celecoxib and rofecoxib effectively prevent PCO formation and aid in controlling postoperative inflammation.

## References

[1] Francis PJ Berry V Moore AT Bhattacharya S Lens biology: development and human cataractogenesis. Trends Genet 1999 15 191 6 10322486 10.1016/s0168-9525(99)01738-2

[2] Davidson MG, Nelms SR. Diseases of the lens and cataract formation. In: Gelatt KN, editor. Veterinary Ophthalmology. 3rd ed. Philadelphia: Lippincott/Williams & Wilkins; 1999. p. 797-825.

[3] Beebe DC. The Lens. In: Kaufman PL, Alm A, editors. Adler's physiology of the eye: clinical application. 10th ed. St. Louis: Mosby; 2003. p. 117-58.

[4] Apple DJ, Rabb MF. Lens and pathology of intraocular lenses. In: Apple DJ, Rabb MF, editors. Ocular Pathology: clinical applications and self-assessment, 5th ed. St. Louis: Mosby; 1998. p. 117-204.

[5] Medvedovic M Tomlinson CR Call MK Grogg M Tsonis PA Gene expression and discovery during lens regeneration in mouse: regulation of epithelial to mesenchymal transition and lens differentiation. Mol Vis 2006 12 422 40 http://www.molvis.org/molvis/v12/a49/16710166

[6] de Iongh RU Wederell E Lovicu FJ McAvoy JW Transforming growth factor-beta-induced epithelial-mesenchymal transition in the lens: a model for cataract formation. Cells Tissues Organs 2005 179 43 55 15942192 10.1159/000084508

[7] Saika S Ikeda K Yamanaka O Sato M Muragaki Y Ohnishi Y Ooshima A Nakajima Y Namikawa K Kiyama H Flanders KC Roberts AB Transient adenoviral gene transfer of Smad7 prevents injury-induced epithelial-mesenchymal transition of lens epithelium in mice. Lab Invest 2004 84 1259 70 15258599 10.1038/labinvest.3700151

[8] Lu S Yu G Zhu Y Archer MC Cyclooxygenase-2 overexpression in MCF-10F human breast epithelial cells inhibits proliferation, apoptosis and differentiation, and causes partial transformation. Int J Cancer 2005 116 847 52 15856465 10.1002/ijc.21142

[9] Huber MA Kraut N Beug H Molecular requirements for epithelial-mesenchymal transition during tumor progression. Curr Opin Cell Biol 2005 17 548 58 16098727 10.1016/j.ceb.2005.08.001

[10] Savagner P Leaving the neighborhood: molecular mechanisms involved during epithelial-mesenchymal transition. Bioessays 2001 23 912 23 11598958 10.1002/bies.1132

[11] McDonnell PJ Zarbin MA Green WR Posterior capsule opacification in pseudophakic eyes. Ophthalmology 1983 90 1548 53 6677855 10.1016/s0161-6420(83)34350-5

[12] Apple DJ Solomon KD Tetz MR Assia EI Holland EY Legler UF Tsai JC Castaneda VE Hoggatt JP Kostick AM Posterior capsule opacification. Surv Ophthalmol 1992 37 73 116 1455302 10.1016/0039-6257(92)90073-3

[13] Sinskey RM Cain W Jr The posterior capsule and phacoemulsification. J Am Intraocul Implant Soc 1978 4 206 7 748313 10.1016/s0146-2776(78)80080-9

[14] Wilhelmus KR Emery JM Posterior capsule opacification following phacoemulsification. Ophthalmic Surg 1980 11 264 7 7383530

[15] Binkhorst CD Gobin MH Injuries to the eye with lens opacity in young children. Ophthalmologica 1964 148 169 83 14214678 10.1159/000304682

[16] Hiles DA Wallar PH Phacoemulsification versus aspiration in infantile cataract surgery. Ophthalmic Surg 1974 5 13 6 4453404

[17] Bras ID Colitz CM Saville WJ Gemensky-Metzler AJ Wilkie DA Posterior capsular opacification in diabetic and nondiabetic canine patients following cataract surgery. Vet Ophthalmol 2006 9 317 27 16939460 10.1111/j.1463-5224.2006.00458.x

[18] Wormstone IM Posterior capsule opacification: a cell biological perspective. Exp Eye Res 2002 74 337 47 12014915 10.1006/exer.2001.1153

[19] Nishi O Posterior capsule opacification. Part 1: Experimental investigations. J Cataract Refract Surg 1999 25 106 17 9888086 10.1016/s0886-3350(99)80020-0

[20] Biswas NR Mongre PK Das GK Sen S Angra SK Vajpayee RB Animal study on the effects of catalin on aftercataract and posterior capsule opacification. Ophthalmic Res 1999 31 140 2 9933777 10.1159/000055526

[21] Cortina P Gomez-Lechon MJ Navea A Menezo JL Terencio MC Diaz-Llopis M Diclofenac sodium and cyclosporin A inhibit human lens epithelial cell proliferation in culture. Graefes Arch Clin Exp Ophthalmol 1997 235 180 5 9085114 10.1007/BF00941726

[22] Seki T Yabe N Kawashima C Long-term efficacy of diclofenac sodium after PEA and IOL surgery. Folia Ophthalmology Japan 1992 43 1452 6

[23] Gately S The contributions of cyclooxygenase-2 to tumor angiogenesis. Cancer Metastasis Rev 2000 19 19 27 11191059 10.1023/a:1026575610124

[24] Williams CS Mann M DuBois RN The role of cyclooxygenases in inflammation, cancer, and development. Oncogene 1999 18 7908 16 10630643 10.1038/sj.onc.1203286

[25] Dubois RN Abramson SB Crofford L Gupta RA Simon LS Van De Putte LB Lipsky PE Cyclooxygenase in biology and disease. FASEB J 1998 12 1063 73 9737710

[26] Marnett LJ DuBois RN COX-2: a target for colon cancer prevention. Annu Rev Pharmacol Toxicol 2002 42 55 80 11807164 10.1146/annurev.pharmtox.42.082301.164620

[27] Thun MJ Henley SJ Patrono C Nonsteroidal anti-inflammatory drugs as anticancer agents: mechanistic, pharmacologic, and clinical issues. J Natl Cancer Inst 2002 94 252 66 11854387 10.1093/jnci/94.4.252

[28] Tan HN Liu Y Diao HL Yang ZM Cyclooxygenases and prostaglandin E synthases in preimplantation mouse embryos. Zygote 2005 13 103 8 16128405 10.1017/s0967199405003187

[29] Cha YI Solnica-Krezel L DuBois RN Fishing for prostanoids: deciphering the developmental functions of cyclooxygenase-derived prostaglandins. Dev Biol 2006 289 263 72 16310177 10.1016/j.ydbio.2005.10.013

[30] Vane JR Bakhle YS Botting RM Cyclooxygenases 1 and 2. Annu Rev Pharmacol Toxicol 1998 38 97 120 9597150 10.1146/annurev.pharmtox.38.1.97

[31] Verburg KM Maziasz TJ Weiner E Loose L Geis GS Isakson PC Cox-2-specific inhibitors: definition of a new therapeutic concept. Am J Ther 2001 8 49 64 11304658 10.1097/00045391-200101000-00009

[32] Mazhar D Ang R Waxman J COX inhibitors and breast cancer. Br J Cancer 2006 94 346 50 16421592 10.1038/sj.bjc.6602942 PMC2361146

[33] Dai Y Wang WH Non-steroidal anti-inflammatory drugs in prevention of gastric cancer. World J Gastroenterol 2006 12 2884 9 16718813 10.3748/wjg.v12.i18.2884 PMC4087805

[34] Marks F Furstenberger G Neufang G Muller-Decker K Mouse skin as a model for cancer chemoprevention by nonsteroidal anti-inflammatory drugs. Recent Results Cancer Res 2003 163 46 57discussion264-6 12903842 10.1007/978-3-642-55647-0_5

[35] Sheng H Shao J Kirkland SC Isakson P Coffey RJ Morrow J Beauchamp RD DuBois RN Inhibition of human colon cancer cell growth by selective inhibition of cyclooxygenase-2. J Clin Invest 1997 99 2254 9 9151799 10.1172/JCI119400 PMC508057

[36] Arico S Pattingre S Bauvy C Gane P Barbat A Codogno P Ogier-Denis E Celecoxib induces apoptosis by inhibiting 3-phosphoinositide-dependent protein kinase-1 activity in the human colon cancer HT-29 cell line. J Biol Chem 2002 277 27613 21 12000750 10.1074/jbc.M201119200

[37] Zhang GS Liu DS Dai CW Li RJ Antitumor effects of celecoxib on K562 leukemia cells are mediated by cell-cycle arrest, caspase-3 activation, and downregulation of Cox-2 expression and are synergistic with hydroxyurea or imatinib. Am J Hematol 2006 81 242 55 16550520 10.1002/ajh.20542

[38] Michael MS Badr MZ Badawi AF Inhibition of cyclooxygenase-2 and activation of peroxisome proliferator-activated receptor-gamma synergistically induces apoptosis and inhibits growth of human breast cancer cells. Int J Mol Med 2003 11 733 6 12736714

[39] Colitz CM Davidson MG McGAHAN MC. Telomerase activity in lens epithelial cells of normal and cataractous lenses. Exp Eye Res 1999 69 641 9 10620393 10.1006/exer.1999.0739

[40] Ramakers C Ruijter JM Deprez RH Moorman AF Assumption-free analysis of quantitative real-time polymerase chain reaction (PCR) data. Neurosci Lett 2003 339 62 6 12618301 10.1016/s0304-3940(02)01423-4

[41] McDonnell PJ Rowen SL Glaser BM Sato M Posterior capsule opacification. An in vitro model. Arch Ophthalmol 1985 103 1378 81 4038131 10.1001/archopht.1985.01050090130047

[42] Kappelhof JP Vrensen GF The pathology of after-cataract. A minireview. Acta Ophthalmol Suppl 1992 205 13 24 1332409 10.1111/j.1755-3768.1992.tb02176.x

[43] Davidson MG Wormstone M Morgan D Malakof R Allen J McGahan MC Ex vivo canine lens capsular sac explants. Graefes Arch Clin Exp Ophthalmol 2000 238 708 14 11011693 10.1007/s004170000158

[44] Behrens J Vakaet L Friis R Winterhager E Van Roy F Mareel MM Birchmeier W Loss of epithelial differentiation and gain of invasiveness correlates with tyrosine phosphorylation of the E-cadherin/beta-catenin complex in cells transformed with a temperature-sensitive v-SRC gene. J Cell Biol 1993 120 757 66 8425900 10.1083/jcb.120.3.757 PMC2119534

[45] Cano A Perez-Moreno MA Rodrigo I Locascio A Blanco MJ del Barrio MG Portillo F Nieto MA The transcription factor snail controls epithelial-mesenchymal transitions by repressing E-cadherin expression. Nat Cell Biol 2000 2 76 83 10655586 10.1038/35000025

[46] Boyd D Invasion and metastasis. Cancer Metastasis Rev 1996 15 77 89 8842480 10.1007/BF00049488

[47] Wormstone IM Tamiya S Anderson I Duncan G TGF-beta2-induced matrix modification and cell transdifferentiation in the human lens capsular bag. Invest Ophthalmol Vis Sci 2002 43 2301 8 12091431

[48] Maidment JM Duncan G Tamiya S Collison DJ Wang L Wormstone IM Regional differences in tyrosine kinase receptor signaling components determine differential growth patterns in the human lens. Invest Ophthalmol Vis Sci 2004 45 1427 35 15111598 10.1167/iovs.03-1187

[49] Gordon-Thomson C de Iongh RU Hales AM Chamberlain CG McAvoy JW Differential cataractogenic potency of TGF-beta1, -beta2, and -beta3 and their expression in the postnatal rat eye. Invest Ophthalmol Vis Sci 1998 39 1399 409 9660488

[50] Lee EH Joo CK Role of transforming growth factor-beta in transdifferentiation and fibrosis of lens epithelial cells. Invest Ophthalmol Vis Sci 1999 40 2025 32 10440257

[51] Hales AM Chamberlain CG Murphy CR McAvoy JW Estrogen protects lenses against cataract induced by transforming growth factor-beta (TGFbeta). J Exp Med 1997 185 273 80 9016876 10.1084/jem.185.2.273 PMC2196117

[52] Majima K Kojima Y Ouhashi F Cell biological analysis with respect to cause of fibrous opacification of the anterior capsule after cataract extraction. Ophthalmologica 1998 212 364 8 9787225 10.1159/000027369

[53] Pandey SK Cochener B Apple DJ Colin J Werner L Bougaran R Trivedi RH Macky TA Izak AM Intracapsular ring sustained 5-fluorouracil delivery system for the prevention of posterior capsule opacification in rabbits: a histological study. J Cataract Refract Surg 2002 28 139 48 11777723 10.1016/s0886-3350(01)01069-0

[54] Zaturinsky B Naveh N Saks D Solomon AS Prevention of posterior capsular opacification by cryolysis and the use of heparinized irrigating solution during extracapsular lens extraction in rabbits. Ophthalmic Surg 1990 21 431 4 2381679

[55] Steinberg EP Javitt JC Sharkey PD Zuckerman A Legro MW Anderson GF Bass EB O'Day D The content and cost of cataract surgery. Arch Ophthalmol 1993 111 1041 9 8352686 10.1001/archopht.1993.01090080037016

[56] Ellwein LB Urato CJ Use of eye care and associated charges among the Medicare population: 1991-1998. Arch Ophthalmol 2002 120 804 11 12049587 10.1001/archopht.120.6.804

[57] Moisseiev J Bartov E Schochat A Blumenthal M Long-term study of the prevalence of capsular opacification following extracapsular cataract extraction. J Cataract Refract Surg 1989 15 531 3 2810087 10.1016/s0886-3350(89)80110-5

[58] Takeda K Kanekura T Kanzaki T Negative feedback regulation of phosphatidylinositol 3-kinase/Akt pathway by over-expressed cyclooxygenase-2 in human epidermal cancer cells. J Dermatol 2004 31 516 23 15492414 10.1111/j.1346-8138.2004.tb00547.x

[59] Wilgus TA Koki AT Zweifel BS Kusewitt DF Rubal PA Oberyszyn TM Inhibition of cutaneous ultraviolet light B-mediated inflammation and tumor formation with topical celecoxib treatment. Mol Carcinog 2003 38 49 58 14502644 10.1002/mc.10141

[60] Vanderveen EE Grekin RC Swanson NA Kragballe K Arachidonic acid metabolites in cutaneous carcinomas. Evidence suggesting that elevated levels of prostaglandins in basal cell carcinomas are associated with an aggressive growth pattern. Arch Dermatol 1986 122 407 12 3456741 10.1001/archderm.122.4.407

[61] Tsujii M DuBois RN Alterations in cellular adhesion and apoptosis in epithelial cells overexpressing prostaglandin endoperoxide synthase 2. Cell 1995 83 493 501 8521479 10.1016/0092-8674(95)90127-2

[62] Buchanan FG Wang D Bargiacchi F DuBois RN Prostaglandin E2 regulates cell migration via the intracellular activation of the epidermal growth factor receptor. J Biol Chem 2003 278 35451 7 12824187 10.1074/jbc.M302474200

[63] Kawamori T Uchiya N Sugimura T Wakabayashi K Enhancement of colon carcinogenesis by prostaglandin E2 administration. Carcinogenesis 2003 24 985 90 12771044 10.1093/carcin/bgg033

[64] Kulp SK Yang YT Hung CC Chen KF Lai JP Tseng PH Fowble JW Ward PJ Chen CS 3-phosphoinositide-dependent protein kinase-1/Akt signaling represents a major cyclooxygenase-2-independent target for celecoxib in prostate cancer cells. Cancer Res 2004 64 1444 51 14973075 10.1158/0008-5472.can-03-2396

[65] Ebert AD Bartley J David M Aromatase inhibitors and cyclooxygenase-2 (COX-2) inhibitors in endometriosis: new questions--old answers? Eur J Obstet Gynecol Reprod Biol 2005 122 144 50 16157442 10.1016/j.ejogrb.2005.04.017

[66] Attar E Bulun SE Aromatase and other steroidogenic genes in endometriosis: translational aspects. Hum Reprod Update 2006 12 49 56 16123052 10.1093/humupd/dmi034

[67] Colitz CMH, Chandler HL, Lu P, Sugimoto Y, Barden CA, Metzler ALG, Wilkie DA, Bras ID, Kuonen VJ, Robbin TE. Estrogen Localization and Local Synthesis of Estrogen by Cataractous LEC. ARVO Annual Meeting; 2005 May 1-5; Fort Lauderdale(FL).

[68] Marshall JL Stanfield KM Silverman L Khan KN Enhanced expression of cyclooxygenase-2 in glaucomatous dog eyes. Vet Ophthalmol 2004 7 59 62 14738509 10.1111/j.1463-5224.2004.04001.x

[69] Sellers RS Silverman L Khan KN Cyclooxygenase-2 expression in the cornea of dogs with keratitis. Vet Pathol 2004 41 116 21 15017024 10.1354/vp.41-2-116

[70] Cullen CL Sims DE Singh A McMarville C Wilcock BP Simmons DL Cyclooxygenase-1, -2, and -3 expression in clinically normal and glaucomatous canine eyes. Vet Ophthalmol 2004 7 441

